# Transcriptional analysis links B cells and TERT expression to favorable prognosis in head and neck cancer

**DOI:** 10.1093/pnasnexus/pgad046

**Published:** 2023-02-10

**Authors:** Su Xian, Magalie Dosset, Andrea Castro, Hannah Carter, Maurizio Zanetti

**Affiliations:** Division of Medical Genetics, Department of Medicine, Bioinformatics and System Biology Program, University of California San Diego, La Jolla, CA 92093, USA; The Laboratory of Immunology, Department of Medicine and Moores Cancer Center, University of California San Diego, La Jolla, CA 92093, USA; Division of Medical Genetics, Department of Medicine, Bioinformatics and System Biology Program, University of California San Diego, La Jolla, CA 92093, USA; Division of Medical Genetics, Department of Medicine, Bioinformatics and System Biology Program, University of California San Diego, La Jolla, CA 92093, USA; The Laboratory of Immunology, Department of Medicine and Moores Cancer Center, University of California San Diego, La Jolla, CA 92093, USA

**Keywords:** B cells, telomerase, tertiary lymphoid structures, transcriptomics, head and neck cancer

## Abstract

Telomerase reverse transcriptase (TERT) is a conserved self-tumor antigen overexpressed in ∼85% of tumor cells and is immunogenic in cancer patients. The effect of TERT expression on the regulation of intratumor adaptive immunity has not yet been investigated. We used RNA sequencing data from The Cancer Genome Atlas (TCGA) in 11 solid tumor types to investigate potential interactions between TERT expression, and B and T cell infiltrate in the tumor microenvironment. We found a positive correlation between TERT expression, B and T cells in four cancer types with the strongest association in head and neck squamous cell carcinoma (HSNCC). In HNSCC a B^high^/TERT^high^ signature was associated with improved progression-free survival (PFS) (*P* = 0.0048). This effect was independent of HPV status and not shared in comparable analysis by other conserved tumor antigens (NYESO1, MUC1, MAGE, and CEA). B^high^/TERT^high^ HNSCC tumors also harbored evidence of tertiary lymphoid structure (TLS) such as signatures for germinal center (GC) and switched memory B cells, central memory CD4 and effector memory CD8 T cells. B^high^/TERT^high^ HNSCC tumors also showed an up-regulation of genes and pathways related to B and T cell activation, proliferation, migration, and cytotoxicity, while factors associated with immunosuppression and cancer cell invasiveness were down-regulated. In summary, our study uncovers a new association between high TERT expression and high B cell infiltrate in HNSCC, suggesting a potential benefit from therapeutic strategies that invigorate intratumor TERT-mediated T-B cooperation.

**Significance Statement**

Current emphasis of cancer immunotherapy is on neoantigens and immune checkpoint inhibitors that rely on the immunogenicity of tumor neoantigens. Telomerase reverse transcriptase (TERT) is a conserved self-tumor antigen overexpressed in the great majority of cancer cells, is immunogenic, and is likely to play a role in immune surveillance. The present study provides evidence that in head and neck squamous cell carcinoma (HNSCC) high TERT expression and high B lymphocyte tumor infiltration are associated with improved progression-free survival (PFS) and distinct activation characteristics of intratumor B and T lymphocytes. The identification of a new signature and immune phenotype associated with the improved clinical outcome has implications for the development of new forms of immunotherapy in HPV-negative HNSCC patients.

## Introduction

Head and neck squamous cell carcinoma (HNSCC) is the sixth most common malignancy; it accounts for 90% of all head and neck cancers (1), has a mortality rate of approximately 50–60% in year one and an overall five-year survival of ∼50% (2). Long-term tobacco use, consumption of alcohol, and infection with high-risk types of human papilloma virus (HPV) are considered the main oncogenic drivers (3). HPV-negative HNSCC patients, which represent the majority of HNSCC tumors, have the worse outcome (4). The treatment of metastatic, unresectable HNSCC consists of chemotherapy, radiation, and immune checkpoint therapy (5, 6) but only a small fraction of patients benefit from immune checkpoint therapy (7, 8).

In recent years the immunology of HNSCC has received considerable attention. As in most cancer types, a high density of T lymphocyte infiltrate correlates with better clinical outcomes. While the contribution of T cells for durable tumor protection has been extensively studied, the exact role of B cells has not been thoroughly interrogated. B cells are best known for their ability to recognize antigen and produce antibodies, but they can also internalize antigen via the B cell receptor (BCR) serving as antigen-presenting cells (APCs). An initial step of the adaptive immune response is T-B cooperation (9), a twofold process where CD4 T cells are activated by B cells and in turn provide cytokines to B cells to support antibody production and isotype switching. Recent reports have analyzed the antibody response of HNSCC tumor against conserved tumor and HPV antigens, respectively (10, 11), also proposing that B cells in HNSCC tumors are part of tertiary lymphoid structures (TLS), i.e. organized aggregates of various types of immune cells that include B and T cells and dendritic cells (DCs) that resemble follicles in secondary lymphoid organs (12, 13). TLS are an increasingly common finding in most cancer types and are often linked with better prognosis (13).

HNSCC tumors have a relatively high mutational burden (TMB) compared to other tumor types (14) and significant expression of APOBEC3B in HPV-positive tumors (15). The AID/APOBEC family of cytidine deaminases is an endogenous source of mutations in many cancers, including HNSCC (16, 17). In particular, APOBEC3 has been reported to be significantly higher in HPV-positive relative to HPV-negative HNSCC tumors (15). However, the TMB in HNSCC tumors tends to show no relationship with tumor neoantigen load (18) and the overall response to immune checkpoint inhibitors is modest (13.3–22%) (8), raising the possibility that local adaptive immune responses in HNSCC tumors involve instead conserved tumor antigens (19). Telomerase reverse transcriptase (TERT) is a component of telomerase, the unique cellular enzyme that synthesizes the tandem 5′-TTAGGG-3′ exonucleotide repeats of telomeric DNA by reverse transcription of its own RNA template (20). Telomerase confers immortality to cells (21) and is a key hallmark of cancer (22). It is overexpressed in >85% of cancer cells and tumors of various histology (23). Since the discovery that TERT, a self-tumor antigen, is immunogenic in cancer patients (24), numerous studies have shown that TERT can elicit both CD8 (25) and CD4 (26) T cell responses. Furthermore, CD8 and CD4 T cells directed against TERT represent an important component of anticancer immunity (27–29). Finally, we previously showed that a B lymphoblastoid cell line presents endogenous TERT (30), demonstrating that B cells can process and present this self-tumor antigen. Altogether, these factors make TERT a candidate link between B and T cells in a cross-talk to initiate adaptive immunity locally.

Here, we used transcriptomic data from The Cancer Genome Atlas (TCGA) to evaluate the prognostic value of TERT and adaptive immune B and T cells in 11 cancer types, including those with accepted HPV pathogenesis and those with prevalent mutations in the TERT promoter region. Compared to B^high^/TERT^low^, a B^high^/TERT^high^ tumor profile was significantly associated with favorable clinical outcome for HNSCC, a benefit independent of the tumor HPV status. Importantly, we revealed that the prognosis of patients with B^high^ tumors was not impacted by the amount of other conserved tumor antigens. Although TERT levels did not associate with the abundance of T cell infiltrate and TLS formation, B^high^/TERT^high^ tumors had an increased proportion of germinal center (GC) B cells, central memory CD4 T cells, and effector memory CD8 T cells. Compared to the other tumor antigens, B^high^/TERT^high^ HNSCC tumors were characterized by a distinct gene expression signature associated with increased B and T cell activation, proliferation, and cytotoxicity. Overall, this study uncovers the singularity of TERT compared to other conserved tumor antigens and the potential importance of a TERT-based B-T cooperation in the generation of an active antitumor immunity providing clinical benefit in HNSCC. The data presented in this report suggest that the B^high^/TERT^high^ phenotype is associated with more favorable clinical outcomes suggesting that new approaches leveraging B cells, TERT, or both could be used to reinforce local antitumor immunity.

## Results

### TERT expression and adaptive immune cell infiltrate predict survival in HNSCC

**Fig. 1.**
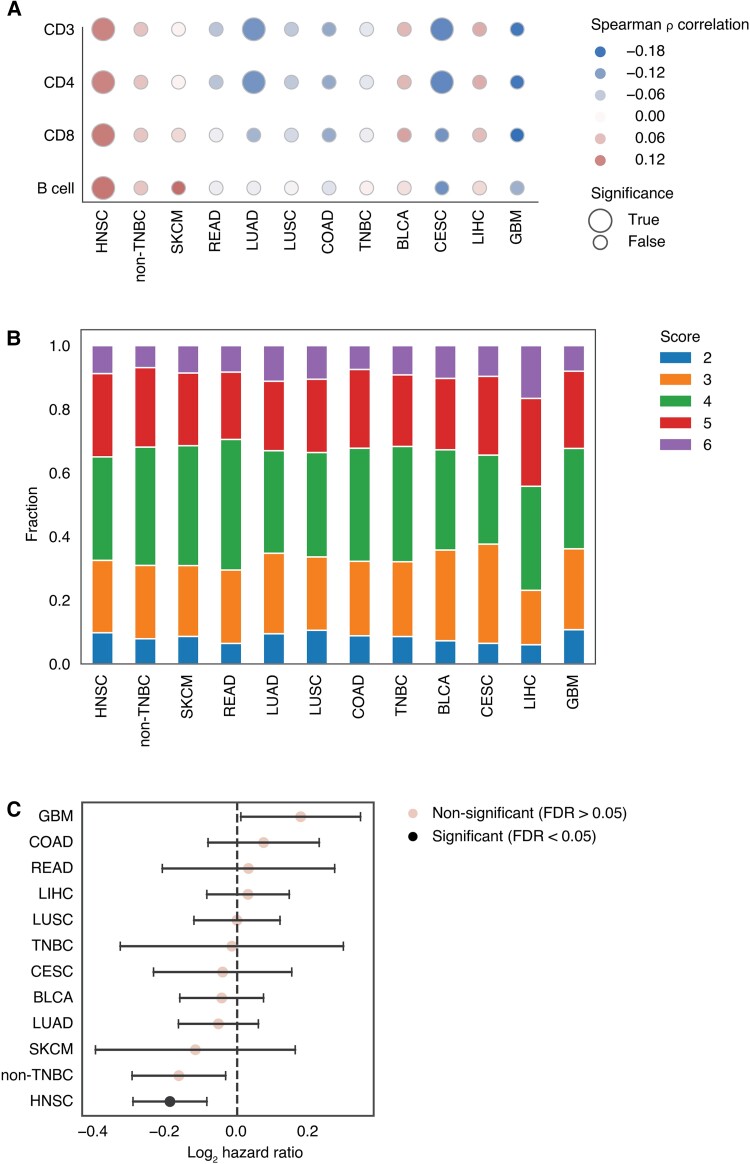
Correlation between TERT and T/B cell infiltrate varies across solid cancers and predicts survival. (A) Spearman correlation between TERT mRNA expression and immune cell infiltration signatures across 11 TCGA tumor types (HNSCC, non-TNBC, SKCM, READ, LUAD, LUSC, COAD, TNBC, BLCA, CESC, LIHC, and GBM. BRCA is split into TNBC and non-TNBC). Statistical significance adjusted using the Benjamini–Hochberg method. (B) Development of the TERT immune score using adaptive immune cells and TERT mRNA expression for the 11 cancer types. An ordinal score ranging from 1 ∼ 3 is assigned for adaptive immune cells and TERT, respectively, using quantile cutoffs (score = 1 for quantile < 0.3, score = 2 for quantile between 0.3 and 0.7, score = 3 for quantile above 0.7). The cancer immune score is the sum of the two ordinal scores. See methods for details. (C) Fraction of tumors receiving a given cancer immune score (ranging from 2 ∼ 6) for the 11 tumor types. (D) Forest plot showing the coefficient and 95% CI of the cancer immune score in a Cox proportional hazard model predicting PFS across 11 tumor types. Statistical significance adjusted by the Benjamini–Hochberg method.

Here, we analyzed tumor RNA sequencing data from TCGA for 4,535 patients representing 11 solid cancer types: bladder (BLCA), breast (divided into triple negative—TNBC—and non-TNBC), cervical (CESC), colorectal (COAD), glioblastoma (GBM), head and neck (HNSCC), liver (LIHC), rectal (READ), skin (SKCM), and lung (LUAD and LUSC) cancers. We found an association between TERT mRNA expression and adaptive immune B and T cell infiltrates in multiple tumor types. TERT expression positively correlated with infiltration of B and T cells (adaptive immune cells) in non-TNBC, BLCA, SKCM, and LIHC cancers, with the strongest association in HNSCC (FDR < 0.05) (Fig. 1A). By contrast, we found an inverse correlation between TERT expression and adaptive immune cells in LUAD, LUSC, GBM, CESC, COAD, and READ (Fig. 1A). To investigate whether these correlations predict progression-free survival (PFS), a cancer immune score was established based on the infiltration of adaptive immune cells and TERT expression level for each cancer type (Fig. 1B). Specifically, we ranked tumors according to TERT expression or adaptive immune cell infiltrate (B and T cell), scored <30%, 30–60%, and >70% quantiles as 1 to 3 respectively for each category according to (31), then summed scores to obtain the cancer immune score (TERT adaptive immune score). Tumors with a score ≤3 preferentially displayed low TERT expression and low adaptive immune cell infiltrate, while tumors with a score ≥5 tended to have both high levels of TERT expression and adaptive immune cell infiltrate. Tumors with an intermediate score of 4 tended to have a mixed phenotype, i.e. median expression of both parameters. The distribution of patients according to this score for each cancer type is presented in Fig. 1C. Although there was no clear relationship between this score and patients’ clinical outcome across the cancer types evaluated in this study (Fig. S1), we found a high score to be significantly associated with improved survival in HNSCC (Fig. 1D). A similar trend was observed in non-TNBC but not in TNBC breast cancer nor in the remaining cancer types (Fig. 1D). Thus, elevated TERT expression levels are associated with high adaptive immune cell infiltrate and this association provides survival benefit in HNSCC. Therefore, subsequent analyses were focused on HNSCC.

**Fig. 2.**
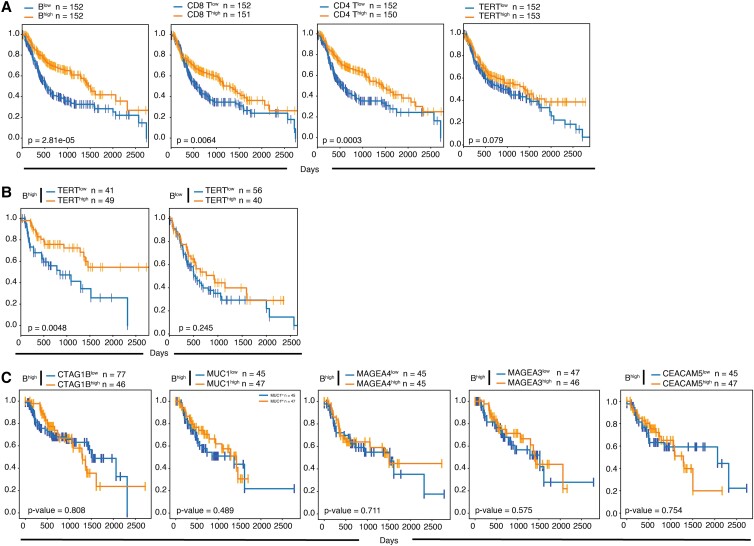
The combination of high-level TERT and B cells markers is associated with better clinical outcomes in HNSCC. (A) PFS analysis in HNSCC stratified by (from left to right) B cell, CD8 T cell, CD4 T cell signature levels, and TERT mRNA expression. (B) PFS analysis in HNSCC expressing low (blue line) or high (orange line) levels of TERT mRNA expression in (left) B^high^ tumors and (right) B^low^ tumors. (C) PFS analysis in HNSCC expressing low (blue line) or high (orange line) mRNA expression levels of other common conserved antigens CTAG1B, MUC1, MAGEA3, MAGEA4, and CEACAM5 (from left to right) in B^high^ tumors.

A univariate PFS analysis revealed that high tumor infiltration of B cells, CD4 T cells, and CD8 T cells was significantly associated with improved PFS (*P* < 0.05), and a similar trend was observed in patients with high TERT expression level (*P* = 0.079) (Fig. 2A). However, in a multivariable Cox proportional hazard model after adjustment for sex, age, stage, HPV status, and TMB as clinical variables, only B cell infiltrate was found to be an independent marker of increased PFS (*P* = 0.02), with a strong trend for TERT expression (*P* = 0.06) (Table 1).

**Table 1.** Cox proportional hazard model for HNSCC.

Patients	log HR	95% CI	*Z*	*P*
Age	0.01	0∼0.03	2.12	**0.03**
Gender	−0.14	−0.47∼0.18	−0.85	0.39
Stage	0.31	0.14∼0.48	3.62	**<0**.**005**
HPV status	−0.36	−0.92∼0.20	−1.27	0.20
TMB (log)	−0.14	−0.34∼0.07	−1.33	0.18
TERT	−0.46	−0.94∼0.01	−1.90	0.06
CD8 T cells	0.36	−0.26∼0.97	1.14	0.25
CD4 T cells	−0.55	−1.35∼0.24	−1.36	0.17
B cells	−0.28	−0.58∼−0.04	−2.27	**0**.**02**

Denoted in bold are P values for covariates that are significantly associated with progression free survival (PFS).

Next, we investigated whether combinations of these two variables can refine the prediction of a patient's prognosis: the combination of high TERT expression (TERT^high^) with high B cell infiltration (B^high^) identified a subset of HNSCC patients with favorable outcome (*n* = 49; B^high^/TERT^high^ group), compared with patients with low TERT expression and high B cells (*n* = 41, B^high^/TERT^low^ group) (Fig. 2B). Of the 10 additional cancer types examined only non-TNBC showed a significant (*P* = 0.044) correlation between B^high^/TERT^high^ and PFS (Fig. S2). When we compared the B^high^/TERT^low^ vs. B^low^/TERT^low^ groups in PFS analysis we found that B cell levels showed no significant difference in survival time (*P* = 0.95) (Fig. S3). Nor did we observe any significant survival association with B cell levels when restricting the analysis to TERT^low^ tumors (KM logrank *P* = 0.954, multivariate Cox PH *P* = 0.66) (Table S1A, B). Among B^high^/TERT^high^ tumors, PFS was independent of tumor mutational burden (TMB) and tumor neoantigen burden in both in TERT^low^ (*P* = 0.70 and *P* = 0.67; Table S1A, B) and TERT^high^ (*P* = 0.75 and *P* = 0.20; Table S1C, D) HNSCC tumors.

There was a substantial difference between the B^high^/TERT^high^ and B^high^/TERT^low^ groups as to the estimated median PFS period. The majority (75%) of B^high^/TERT^high^ patients were still alive after ∼3.5 years in contrast to 30% in the B^high^/TERT^low^ group (*P* = 0.0048). The effect on survival was still apparent and significant (*P* = 0.0406) when splitting the cohort on the median instead. Importantly, TERT expression levels did not influence the outcome of patients with low B cell infiltrate (*P* = 0.245) (Fig. 2B), suggesting that the presence of B cells in the tumor microenvironment is a necessary complement to TERT overexpression in providing clinical benefit. Of note, benefit by TERT in B ^high^ tumors was also observed in non-TNBC breast cancer (*P* = 0.044) whereas a trend toward the opposite effect on clinical course was found in GBM (*P* = 0.165) (Fig. S2). The prognostic impact of the B^high^/TERT^high^ signature was not influenced by the disease stage. When examined by tumor stages (grouping stage I–II and stage III–IV, respectively the B^high^/TERT^high^ signature showed a significant difference in early-stage group (*P* = 0.024), and a near significant trend in the late-stage group (*P* = 0.065) (Fig. S4).

### TERT confers a unique survival benefit compared to other conserved tumor antigens

We determined whether the association between B cell infiltrate and TERT expression (B^high^/TERT^high^ phenotype) was specific or was shared by other conserved tumor antigens previously documented in HNSCC tumors (32). Noticeably, overexpressed NYESO1 (CTAGB1), MUC1, MAGEA3, MAGEA4, or CEA (CEACAM5) antigens did not correlate with TERT improved PFS of patients with high B cell infiltrate (Fig. 2C), suggesting a selective role of TERT over other common tumor antigens in driving local antitumor immunity. We also performed a multivariable Cox proportional hazard analysis for each antigen independently. With the exception of MAGEA3 that showed a barely significant *P*-value (*P* = 0.05), no other conserved tumor antigen reached significance. As to MAGEA3, the effect size was very small (coefficient = −0.01), indicating a very weak effect toward increased survival (Table S2).

### The prognostic value of intratumor B^high^/TERT^high^ expression is independent of HPV status

**Fig. 3.**
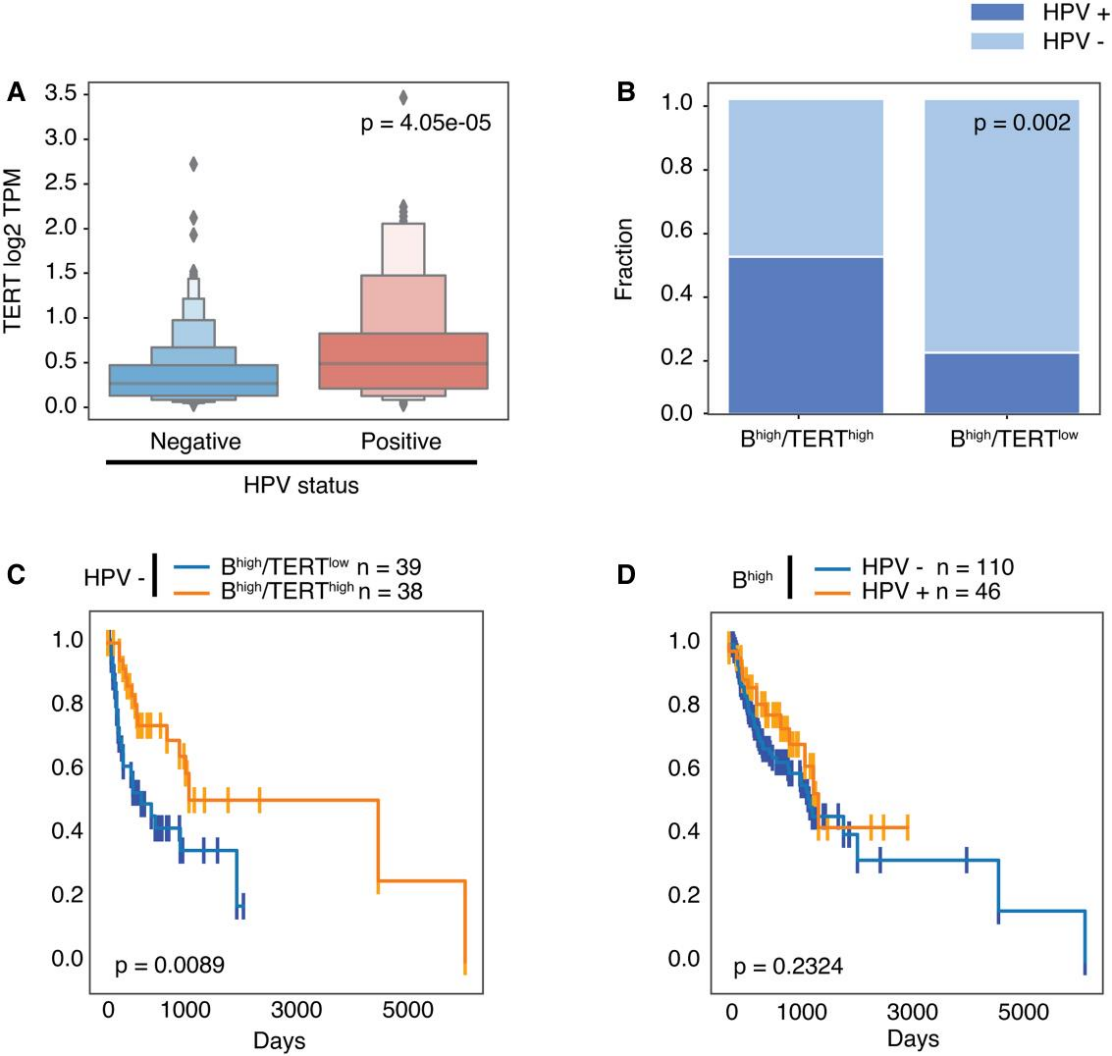
The prognostic value of intratumor B^high^/TERT^high^ expression is independent of the HPV status. (A) Log2 TPM expression of TERT in HPV + and HPV-samples in HNSCC. (B) Percentage of HPV + samples in B^high^/TERT^high^ (*n* = 52) and B^high^/TERT^low^ (*n* = 41) groups. Fisher's exact test. (C) PFS analysis in HNSCC HPV-negative (HPV-) samples, stratified by B^high^/TERT^high^ vs. B^high^/TERT^low^. (D) PFS analysis in B^high^ HNSCC comparing HPV-samples (*n* = 110) vs. HPV+ samples (*n* = 46).

The overall prevalence of HPV in HNSCC ranges from 25 to 35% (33, 34). HPV and its expression have been reported to drive TERT promoter activation (35). As expected, a significant positive correlation was found between TERT expression and HPV status (*P* < 0.001) (Fig. 3A). Fifty percent (50%) of B^high^/TERT^high^ patients were HPV-positive vs. ∼20% in the B^high^/TERT^low^ group (odds ratio = 4.13; *P* = 0.002) (Fig. 3B), consistent with the induction of TERT by the E6 protein expressed in HPV + HNSC tumor cells. To assess whether clinical benefit associated with higher TERT expression was attributable to HPV positivity, we repeated the survival analysis of the HNSCC cohort after removing HPV-positive cases. Surprisingly, the positive impact of TERT expression on favorable PFS persisted (*P* = 0.0089), suggesting that the benefit of elevated TERT expression in the B^high^ group is independent of HPV status (Fig. 3C). In these tumors, higher TERT expression may be due to promoter mutations or other HPV-independent mechanisms (36). Of note, although HPV has been considered a factor of good prognosis in HNSCC (37), we did not observe a significant difference in outcome between B^high^/HPV-positive and B^high^/HPV-negative patients in this cohort (*P* = 0.2324) (Fig. 3D). Altogether, these data suggest that TERT up-regulation in HNSCC tumors may not be uniquely driven by HPV positivity, and that other factors may contribute to TERT expression levels in B^high^ tumors.

### B^high^/TERT^high^ status is associated with increased TLS markers in HNSCC

To better define the role of B^high^/TERT^high^ status from a local immunodynamic standpoint we used gene expression data to assess the potential involvement of TLS, which are organized comprising T-cell and B-cell areas that arise in the context of chronic inflammation and mediate local antigen-driven responses (12, 38). They occur in the TME of numerous solid cancer types and gene signatures of TLS have been used as a proxy for spatial imaging-based analysis of cell infiltrates. The presence of TLS and found to correlate with a good prognosis in most cancers including HNSCC (13).

**Fig. 4.**
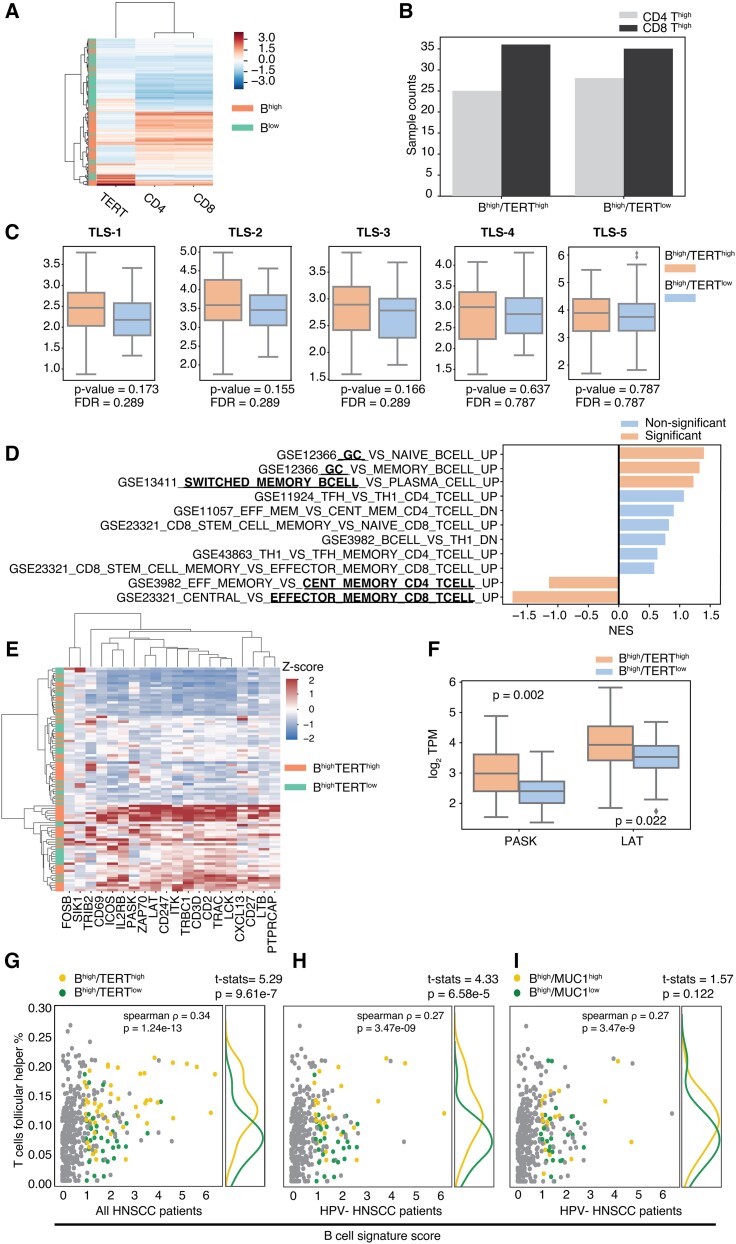
Correlation of high level of TERT expression and B cells with increased TLS-associated markers. (A) Heatmap showing expression of TERT, CD4 T cell, and CD8 T cell markers (see Data stratification and cell marker in Material and Methods) in B^high^ vs. B^low^ HNSCC tumors. (B) Levels of expression of CD4 and CD8 T cell infiltrate in B^high^/TERT^high^ and B^high^/TERT^low^ groups. (C) TLS signature in B^high^/TERT^high^ and B^high^/TERT^low^ tumors using five signatures from four distinct publications (see Table S1). (D) Barplot of GSEA analysis result revealing differences in B cell and T cell signatures between B^high^/TERT^high^ and B^high^/TERT^low^ tumors. (E) Heatmap of TFH signature genes from CIBERSORT LM22 across all B^high^ tumor samples. B^high^/TERT^high^ and B^high^/TERT^low^ status are indicated by the left colorbar. (F) Boxplot of two TFH-associated genes (PASK and LAT) expressed in B^high^/TERT^high^ and B^high^/TERT^low^ groups (G-I). Scatter plots showing correlation between TFH percentage infiltrate (calculated using CIBERSORT) and B cell markers in B^high^/TERT^high^ and B^high^/TERT^low^ tumors from (G) the whole HNSCC cohort, and (H) HPV-negative HNSCC patients. (I) Same as in (H) for B^high^/MUC1^high^ and B^high^/MUC1^low^ in HPV-tumors.

First, we found that HNSCC tumors with high B cell infiltration also displayed high expression of CD4 and CD8 T cell markers (Fig. 4A). However, since no difference in CD4 and CD8 T cell expression levels was found in the B^high^/TERT^high^ group vs. the B^high^/TERT^low^ group (Fig. 4B), it appears as if TERT overexpression is not a determinant of intratumor T cell infiltration. Quantification of TLS based on five distinct published gene signatures (Table S3) (13, 39–41) showed consistent results with a moderate increase in B^high^/TERT^high^ vs. B^high^/TERT^low^ tumors irrespective of the gene signature utilized (Fig. 4C). However indicative, a small increase may be interpreted to suggest that while TLS organization results from a variety of antigen-independent local factors (42), TERT mediates the activation of B and T cells that are organized into TLS.

To better characterize differences in cell population in B^high^/TERT^high^ vs. B^high^/TERT^low^ HNSCC tumors, we performed gene set enrichment analysis (GSEA) on 11 gene sets from the Molecular Signatures Data Base Immunological signatures (C7) that are associated with B and T cell differentiation (Fig. 4D) (43). The analysis revealed a statistically significant enrichment for germinal center (GC) and switched memory B cell features in B^high^/TERT^high^ tumors, which are typically found in mature TLS (13). Consistent with this observation, we found that B^high^/TERT^high^ up-regulated the expression of AICDA (AID) (Fig. S5A), an essential driver of immunoglobulin somatic hypermutation (44). Furthermore, CD4 T cells were strongly associated with a central memory signature, while CD8 T cells rather displayed a profile described for effector memory. We also investigated a T follicular helper (TFH) signature based on the expression of the top 20 markers derived from the CIBERSORT LM22 signature (39) (Fig. 4E). Although no significant difference between B^high^/TERT^high^ and B^high^/TERT^low^ tumors could be identified, two TFH signature-derived genes PASK (PAS domain-containing serine/threonine-protein kinase) and LAT (Linker Activation for T cells) were significantly up-regulated in the B^high^/TERT^high^ group (*P* = 0.002 and *P* = 0.022, respectively) (Fig. 4F). PASK is a kinase involved in glycolysis, a primary source of energy in effector T cells, while LAT is a transmembrane protein part of the TCR complex that is activated in response to antigen stimulation. Therefore, TERT overexpression in B^high^ HNSCC tumors hallmarks pronounced antigen-specific activation of T cells. Furthermore, we found a strong positive correlation between B cells and TFH in B^high^/TERT^high^ vs. B^high^/TERT^low^ tumors (*P* = 9.61e^−7^). This correlation was also independent of HPV status (Fig. 4G, H) and was not observed, for example, in B^high^/MUC1^high^ vs. B^high^/MUC1^low^ tumors (Fig. 4I). This confirms the preferential involvement of TERT as a catalyst of T-B cooperation in HNSCC.

Taken together, these data indicate that high TERT expression in HNSCC tumors with high adaptive immune cell infiltrates may promote the formation of more mature TLS and the differentiation of antigen-specific memory T cells.

### The B^high^/TERT^high^ signature is associated with adaptive immune response gene expression

**Fig. 5.**
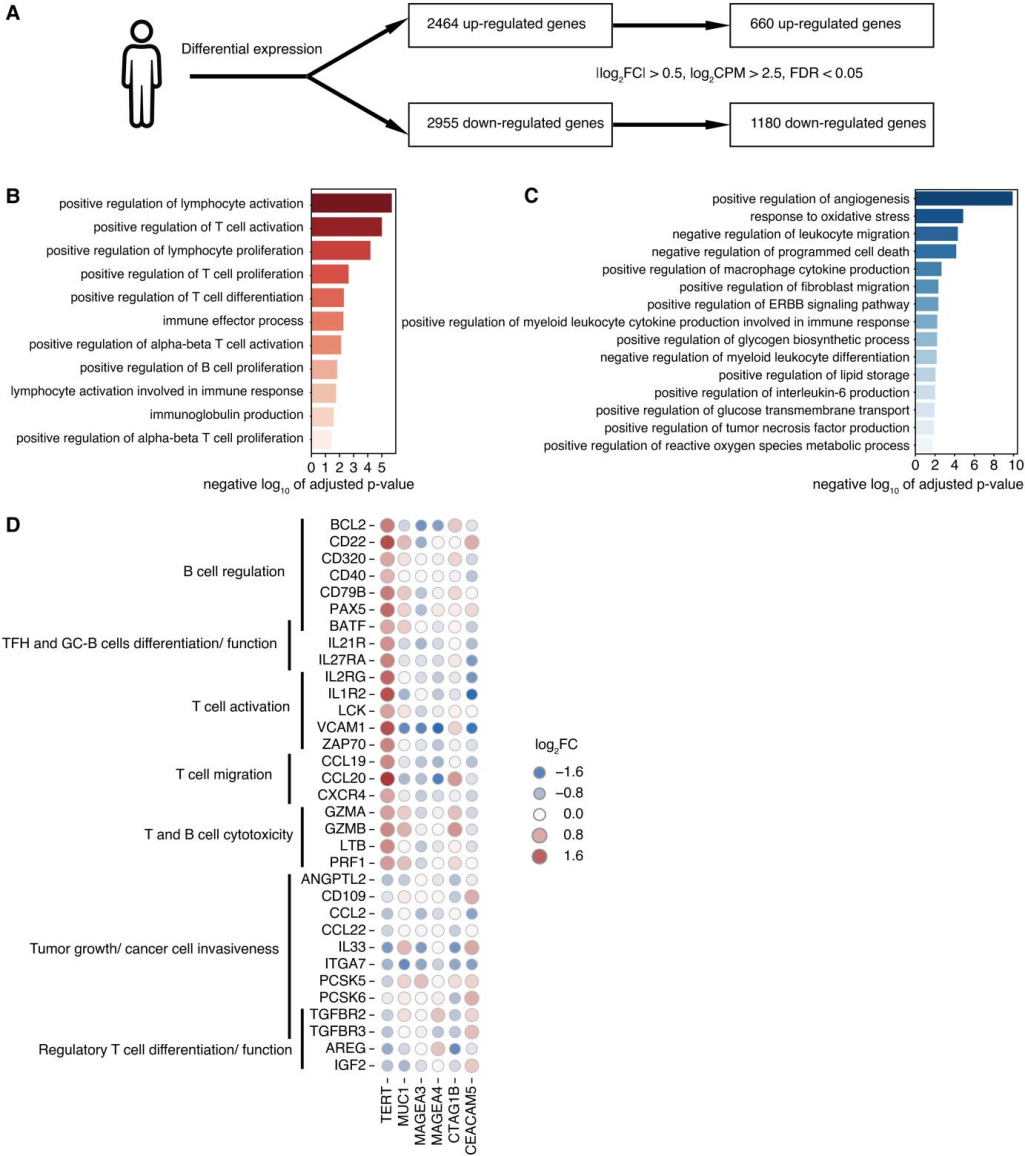
Differential expression analysis between B^high^/TERT^high^ and B^high^/TERT^low^ reveals increased gene expression in the adaptive immune response. (A) Illustration of the differential expression analysis in HNSCC between B^high^/TERT^high^ and B^high^/TERT^low^. (B) and (C) Barplot showing immune-related gene sets evaluated by GO enrichment analysis for (B) 660 up-regulated genes and (C) 1180 down-regulated genes after thresholding using criteria from A). (D) Heatmaps showing the expression of genes involved in immune functions and tumorigenesis across six different conserved antigens (TERT, MUC1, MAGEA3, MAGEA4, CTAG1B, CEACAM5).

To gain insight into the benefit of TERT in HNSCC, we investigated genes differentially regulated in the B^high^/TERT^high^ group compared to the B^high^/TERT^low^ group. Elevated expression levels of TERT in tumors with high B cell infiltrate drove the up-regulation of 2,464 genes and the down-regulation of 2,955 genes, respectively (Fig. 5A). Pathways analysis of the significantly up- and down-regulated genes (FDR < 0.05) revealed that this profile was associated with the induction of genes related to activation and proliferation of B and T cells, and immunoglobulin production (Fig. 5B). Conversely, we found that pathways related to regulation of angiogenesis, glucose transport, lipid storage, and macrophage/myeloid cytokine production were down-regulated (Fig. 5C). Specifically, we identified a cassette of genes differentially (|log_2_FC| > 0.5, log_2_CPM > 2.5, FDR < 0.05) regulated in the B^high^/TERT^high^ group. High-level intratumor TERT expression was associated with increased expression of genes involved in B cell regulation (CD22, CD40, CD79B, PAX5), T cell activation (IL2RG, IL1R2, LCK, VCAM1, ZAP70), TFH and GC-B cell function (BATF, IL21R, IL27), lymphoid organogenesis (LTB), and T cell cytotoxicity (GZMA, GZMB), PRF1 (Fig. 5D). In contrast, we observed decreased expressions of several genes related to regulatory T cells (Treg) differentiation and function (IGF2, TGFBR2, TGFBR3), and cancer cell invasiveness (ANGPTL2, IL33, ITGA7, PCSK5, PCSK6, PTPRB, TBX3, TGFBR2, TGFBR3) (Fig. 5D). Interestingly, this distinct pattern was not observed in B^high^/MUC1^high^, B^high^/NYESO1^high^, B^high^/MAGE^high^ or B^high^/CEA^high^ tumors (Fig. 5D), suggesting TERT may be preferentially involved in stimulating specific antitumor immunity in the microenvironment of HNSCC tumors.

Altogether these data revealed that TERT confers a unique transcriptional profile associated with good prognosis in HNSCC tumors with high adaptive immune cell infiltrate, suggesting that TERT may mediate intratumor T-B cooperation for the generation of active and effective local antitumor immunity.

## Discussion

Here we report an association between high TERT expression and high levels of B cell tumor infiltrate and favorable PFS in HNSCC. Since no other conserved tumor antigen tested yielded a significant positive correlation, a plausible conclusion is that TERT is preferentially engaged in the activation of adaptive immune cells in the HNSCC tumor microenvironment. An activated phenotype of intratumor B lymphocytes in HNSCC tumors is consistent with a previous report (10). This report showed higher expression of two conserved tumor antigens (MAGEA1 and MAGEA3) in HPV-negative tumors and a significant decrease of TP53 in HPV-positive tumors, but no attempt was made to link together these antigens, nor to assess the degree of B cell tumor infiltrate and clinical benefit. Here we show that survival benefit is predicted by high B cell tumor infiltrate together with high TERT expression, but not tumor mutational burden or neaoantigen burden, placing TERT as a key antigen in mediating local antitumor immunity and favorable clinical outcome in HNSCC. This is not surprising given the role of TERT in oncogenesis (45, 46) and its expression at every stage of tumor differentiation (47). The present study points, therefore, to a potential new role for TERT in orchestrating local antitumor immunity via T-B cooperation.

The reason why TERT may be presented more effectively than other antigens in HNSCC increasing PFS may have a twofold explanation: (a) the mechanism of TERT reactivation and expression in HNSCC, and (b) the local immunodynamics involving B cells and TLS formation. The TERT promoter is the most important regulatory element of telomerase expression (48). With few exceptions, TERT is repressed in normal somatic tissues by negative regulators (e.g. p53, RB, WT1, and Mad1) but is reactivated in 85% of human cancers, representing the rate-limiting step in tumorigenesis. Canonical activators of the (wild type) TERT promoter include among others the transcription factor Sp1, the oncogene c-Myc, and the HPV-16 E6 protein (49, 50). TERT reactivation can also reflect chromatin and DNA methylation (51) and chromatin interaction of the TERT promoter with T-INT2 (Tert INTeracting region 2) through elevated levels of β-catenin and the transcription factor JunD (52). β-catenin was previously shown to bind to the TERT promoter regulating TERT expression (53) and also be up-regulated by endoplasmic reticulum stress (54).

In HNSCC, TERT promoter mutations are moderately frequent (range 17–32%) (55, 56) compared to melanoma, glioblastoma, and bladder cancer (57), but more frequent in recurrent vs. non-recurrent squamous cell carcinoma of the oral cavity (58). TERT promoter mutations create *de novo* binding sites for ETS transcription factors (59), among which GABPA selectively binds and activates a mutated TERT promoter, alone (60) or in association with p52 (61), a member of the NF-κB family. GABPA binding to a mutated TERT promoter mediates long-range chromatin interactions that further drive TERT transcription (62). Other factors may be implicated in the reactivation of TERT in HNSCC leading to heightened expression. One is a tissue-determined cytodynamic in combination with TERT promoter mutations when cancer stem cells differentiate into mature cancer cells (63), a phenomenon that would favor TERT reactivation and high expression in tissues with high proliferative/regenerative capacity such as the oral cavity (64). The other is that the TERT gene in HNSCC has been reported to have an increased copy number which would lead to heightened TERT transcription and expression compared to a single-copy gene (65).

Local immunodynamic conditions in HNSCC may favor TERT presentation by B cells to T cells (T-B cooperation). The oropharynx is rich in lymphoid structures known as the Waldeyer's ring which consists of palatine tonsils, nasopharyngeal tonsils, lingual tonsils, and tubal tonsils. Proximity to lymphoid tissue may facilitate trafficking and antigen presentation, steps of critical importance in the interpretation of our findings. A validation of this hypothesis was recently provided in a mouse model of HNSCC in which it was shown that surgical ablation of draining lymphatics eliminated the tumor response to immune checkpoint blockade, worsening overall survival (66).

TCGA data did not permit an assignment of TERT promoter status, leaving unanswered the question as to whether the preferential role of TERT over other antigens and the PFS advantage provided by the B^high^/TERT^high^ signature reflect TERT reactivation and expression via wild-type or mutated promoter. Future experiments addressing the complexity of TERT reactivation and expression in HNSCC, and the role of local immunodynamics involving B cells, will be relevant to understand why TERT and the B^high^/TERT^high^ signature have a positive impact on PFS in HNSCC.

One could argue that the high levels of TERT accounting for the B^high^/TERT^high^ signature could be contributed by B cells, implying the presentation of endogenous TERT by B cells. This would rule out any contribution of TERT promoter mutations in the phenomenon, and restrict its interpretation to the activation of intratumor B cells by other antigens, with little role for local immunodynamic. TERT is transiently expressed in germinal center (GC) B cells (centroblasts and centrocytes) during activation by dendritic cells but not in naïve or memory B cells (67–69). Even a transient induction requires the engagement of the B cell receptor together with costimulatory signals and IL-4 (69). Notwithstanding the fact that GC-like B cells in TLS have been reported (70), and our data point to TLS formation in HNSCC, centroblasts may represent a minority within a population of tumor-infiltrating B cells. Since memory B cells can present antigen and engage in T-B cooperation (70) without population expansion, there would be no necessity to reactivate TERT. An additional consideration is the amount of endogenous TERT transcribed and expressed by activated B cells may be not only transient but also limited quantitatively. For instance, we reported that activated primary B cells are not lysed by autologous TERT-specific CD8 T cells, suggesting poor generation of peptides from endogenous source TERT (71). Our data do not support the view of B cells being the source of TERT in HNSCC. Using CIBERSORTx to deconvolute the relative proportion of cells in the tumor microenvironment (Table S4) (https://dice-database.org/genes/TERT) (72) and by interrogating single-cell data set of an HNSCC cohort (GSE103322) (Fig. S8), we found little if any evidence for TERT expression in B cells arguing that most TERT expression in HNSCC tissues may be contributed predominantly or solely by tumor cells.

A surprising finding of our study is that the B^high^/TERT^high^ signature is apparently not dependent on HPV positivity. In HNSCC the overall prevalence of HPV is 25 to 35% (33, 34). and HPV positivity exhibits an almost complete mutual exclusivity with activating mutations in the TERT promoter and mutations in other known driver genes such as *TP53*, and *CDKN2A* (15). HPV-positive tumors display a significant increase in macrophages and TFH cells (15). Recent reports showed that HPV-positive HNSCC tumors are enriched for B lymphocytes with GC signature and spatial organization of immune cells consistent with TLS formation (40). Similarly, Wieland et al. (11) reported that HPV-positive HNSCC tumors harbor HPV-specific antibody-secreting cells (ASC) in GC-like spatial organization. Thus, while these reports point to a prevalent role of HPV, our data based on an unbiased analysis point to a facilitating role of TERT that is apparently independent of HPV status.

The AID/APOBEC family of cytidine deaminases is an endogenous source of mutations in many cancers, including HNSCC (16, 17). In particular, APOBEC3 has been reported to be significantly higher in HPV-positive relative to HPV-negative HNSCC tumors (15). Unexpectedly, compared to B^high^/TERT^low^ HNSCC tumors, we found that the B^high^/TERT^high^ profile was significantly associated with increased expression of AICDA (*P* = 0.010, log2FC = 0.585) and APOBEC3B (*P* = 0.017, log2FC = 0.476) in HPV-negative HNSCC tumors (Fig. S5B). An increase in AID/APOBEC (log_2_FC = 0.807 and 1.85, respectively) was also observed in B^high^/TERT^high^ HPV-positive tumors albeit not significantly, likely due to the small sample size. Thus, the association with TERT expression in both HPV-positive and HPV-negative patients suggests that AID/APOBEC up-regulation in HPV-negative tumors with the B^high^/TERT^high^ signature may be due to other factors. As noted above, we found no correlation with PFS and either tumor mutational burden or neoantigen burden in either TERT^low^ or TERT^high^ HNSCC tumors.

A transcriptional interrogation using five different TLS signatures pointed to the possibility that in our analysis, like in previous reports (11, 40), tumor-infiltrating B cells are part of TLS formation. However, since we did not image their spatial organization, it cannot be firmly concluded if TLS in the HNSCC tumors studied herein are dispersed (immature) or structured (mature). Our results are consistent with the positive role played by TLS in various cancer types (13) including HNSCC (11, 40). Self-organized intratumor TLS are key tissue-reactive immune events facilitating interactions between adaptive immune cells (12). Emphasis on B cells is also consistent with the conclusions of explorations on antigen presentation within TLS structures showing that antigen presentation is determined only in part by the uptake of soluble antigen by dendritic cells (73).

An immunological consequence of B cell interactions with other immune cells within TLS is T-B cooperation (9), a key initial step in antigen-specific adaptive immune responses. Since CD4 T cells take also part in the activation of CD8 T cells (74) and their long-lasting maintenance in vivo (75, 76), it stands to reason that B cells could play a pivotal role in orchestrating intratumor antitumor immunity in HNSCC. Since intratumor B cell density restricts Tregs in lung carcinoma (77), it is tempting to propose that the B^high^/TERT^high^ signature in HNSCC tumors may provide a twofold advantage: a coordinated induction of local adaptive antitumor immunity, and restriction of Tregs that could down-regulate adaptive T cell responses (78). Contrary to the idea that antigen may drive the formation of TLS (79), our data support the view that TERT overexpression in HNSCC tumors may not be solely responsible for the formation of TLS since a B^high^/TERT^high^ signature drives comparable TLS scores in other tumor types (Fig. S6). Future studies will need to confirm the extent to which B cells present TERT and activate CD4 helper T cells, reshaping the immune response in the tumor microenvironment.

The prognostic value of the B^high^/TERT^high^ profile differed among various cancer types and was associated with improved PFS only in HNSCC and non-TNBC breast cancer. Except for GBM and READ, most B^high^/TERT^high^ tumor types had comparable TLS signature levels (Fig. S6). This argues in favor of factors/mechanisms that oppose the positive effect(s) of the B^high^/TERT^high^ signature in other cancer types (Fig. S7A). For instance, we observed that unresponsive tumors up-regulated several factors associated with immunosuppression and/or cancer cell invasiveness (Fig. S7A and B). Among them, IGF2 (insulin-growth factor 2) and PCSK9 (Proprotein convertase subtilisin/kexin type 9) stand out. IGF signaling and high levels of IGF2 binding proteins (IGFBPs) have been implicated in cancer promotion (80–83). PCSK9 that is overexpressed in human cancers was shown to suppress antitumor T cell immunity (84).

In summary, the scenario suggested by our data is that B cells with the surface receptor for TERT internalize TERT and process/present it to CD4 T cells (T-B cooperation) setting in motion an intratumor adaptive immune response. Since TERT is expressed in the vast majority of tumor cells this intratumor mechanism would result in immunity against cancer cell growth and local invasion. This may explain why the B^high^/TERT^high^ signature is associated with favorable PFS in HNSCC. Our data also suggest new therapeutic approaches leveraging B cells, TERT, or both to heighten intratumor antitumor immunity in HNSCC. However, a “B cell only” approach may not be sufficient without specific instruction by antigen (TERT), and the induction of “TERT immunity alone” may also not be sufficient if B cells are not engaged. Antibodies to TERT in HNSCC patients have not been previously reported, arguing against their protective role by either antibody-dependent cell-mediated cytotoxicity, antibody-dependent cellular phagocytosis, or complement-dependent cytotoxicity. Instead, new therapeutic approaches that invigorate TERT-mediated T-B cooperation using, for example, autologous B lymphocytes engineered to express TERT, could represent the next frontier in the immunotherapy of HNSCC complementing existing T cell-centered immunotherapies (85).

## Materials and methods

### Data stratification and cell marker

We performed stratification using the standards of High (higher than 70% quantile), Medium (within 30–70% quantile), and Low (less than 30% quantile) expression levels for the variables of interest. For two variables, such as B cell High/TERT High (indicated as B^high^/TERT^high^), we took the intersection of samples that satisfied both criteria. For immune cells (B cells, CD4 T cells, CD8 T cells), levels were quantified using the geometric mean of log2TPM of defined cell markers: CD19 and MS4A1 for B cells; CD3D, CD3E, CD3G, CD4 for CD4 T cells; CD3D, CD3E, CD3G, CD8A and CD8B for CD8 T cells.

### Cancer immune score development

The cancer immune score is developed using a sum of the adaptive immune cell (B, CD4, and CD8 T cells) and TERT TERT log2TPM expression levels. The adaptive immune cell and TERT level of expressions are stratified into scores of 1 (less than 30% quantile), 2 (within 30% ∼ 70% quantile), and 3 (higher than 70% quantile). The sum of the adaptive immune stratified score and TERT stratified score (ranging from 2 to 6) is called the cancer immune score.

### Kaplan–Meier survival analysis and cox proportional hazard model

Survival analysis was done using the lifeline packages, version 0.26.4, and python version 3.8.5. For the Cox proportional hazard analysis, age at diagnosis, sex, stage, HPV status, and log2(TMB) were included as clinical covariates in a model evaluating the impact of TERT and adaptive immune infiltrates (CD8 T cells, CD4 T cells, and B cells) on PFS. HPV status was encoded as 1 for positive and 0 for negative. Tumor stage was ordinal scaled from 1 to 4. The PFS data is obtained from (86) in the supplemental information session.

### mRNA expression data and CIBERSORT

The TCGA files were downloaded from the GDC portal on 12/27/2017, using gdc-client v1.3.0. TCGA RNA-seq alignment files were reprocessed using Sailfish software version 0.7.4 (87) to obtain TPM values using the GRCh38 reference genome with default parameters, including all read sequence duplicates. CIBERSORT immune cell infiltrate estimates were generated using the TCGA HNSCC mRNA expression data as processed and used in Xian et al. (31) with all default parameters (LM22 signature). The deconvolution process to infer immune cell gene expression was performed using CIBERSORTx applied to the TCGA HNSCC mRNA expression data under the Impute Cell Expression function. The LM22 signature is merged for 10 major subsets by CIBERSORTx, with all default parameters.

### GSEA analysis for immunologic genesets

Gene set enrichment analysis (GSEA) is done using GSEApy packages, version 0.9.5, in python. Two phenotypes are compared, B^high^/TERT^high^ vs. B^high^/TERT^low^ (see stratifications section for definition). The GSEA analysis was performed using mostly default parameters, as described below. Parameters were set as permutation_type: “gene_set”, permutation_num: 500, method: “t_test”. The genesets were downloaded from msigDB (88) C7 immunologic genesets (http://www.gsea-msigdb.org/gsea/msigdb/genesets.jsp?collection=C7).

### Differential expression analysis

Differential expression analysis was performed using the edgeR package, version 3.32.1(R version 4.0.5). The RNAseq HT-seq read count file was downloaded from TCGA using the gdc-client on 09/11/2019 using the manifest file for all tumor types available. Differential expression analysis was performed for HNSCC comparing B^high^/TERT^high^ and B^high^/TERT^low^ tumors. We then focused on the up-regulated genes and down-regulated genes (FDR < 0.05) that have certain levels of expression (log_2_CPM > 2.5) and a modest fold change (|log_2_FC| > 0.5, which corresponds to a 1.44 fold change in either direction).

### GO annotation of significantly up- and down-regulated genes

Gene ontology (GO) enrichment analysis was performed using an online platform called g:Profiler (89). Multiple-testing correction is performed using the Benjamini–Hochberg FDR method. We focused only on the Biological Process (BP) component.

### Tumor mutational burden and tumor neoantigen burden

The tumor mutational burden counts and estimate of neoantigen burden were calculated as described in (90).

### TLS signature computation

Five distinct TLS gene signatures were collected from the literature (Table S1) (13, 34–36). For each signature, the TLS score was calculated as the arithmetic mean of the log_2_ TPM of the gene list. TLS score distributions were compared using the Wilcoxon rank-sum test. The TLS score used in Fig. S4 was computed using the 12 cytokine genes, with the application of single-sample GSEA (ssGSEA), according to (36).

### HNSCC HPV status

The clinical indication of HPV status was downloaded from cBioPortal (91), from the analysis of Head and Neck Squamous Cell Carcinoma (TCGA, PanCancer Atlas) (https://www.cbioportal.org/study/clinicalData?id=hnsc_tcga_pan_can_atlas_2018).

### Software packages and version

Spearman correlation analysis and Fisher's exact test were performed using the scipy package, version 1.7.3. Multiple-testing correction was performed using the stats.multitest.multipletests function from Statsmodels 0.12.0. All analysis (except differential expression analysis mentioned above using R) was done using python, version 3.8.5.

### Data and materials availability

Bioinformatic data have been deposited at https://github.com/cartercompbio/TERT_adaptive_immune_score

## Supplementary Material

pgad046_Supplementary_Data

## Data Availability

The results shown here are based upon data generated by the TCGA Research Network: https://www.cancer.gov/tcga. The data are available at https://portal.gdc.cancer.gov/.
