# Supplementary material for: Transcriptional analysis links B cells and TERT expression to favorable prognosis in head and neck cancer
Source: PNAS Nexus. 2023 Feb 10;2(3):pgad046. doi: 10.1093/pnasnexus/pgad046 (PMC10003760; doi:10.1093/pnasnexus/pgad046)
Supplement: pgad046_Supplementary_Data [file pgad046_supplementary_data.pdf]

Supporting Information for
Transcriptional analysis links B cells and TERT expression to favorable prognosis in head and neck cancer

Su Xian[1†], Magalie Dosset[2†], Andrea Castro[1], Hannah Carter[1*] and Maurizio Zanetti[2*]

[†] S.X. and M.D. contributed equally to this work
* Corresponding authors. M.Z. and H.C. Email: mzanetti@health.ucsd.edu, hkcarter@health.ucsd.edu

This PDF file includes:

Figures S1 to S8
Tables S1 to S4

Supporting Information

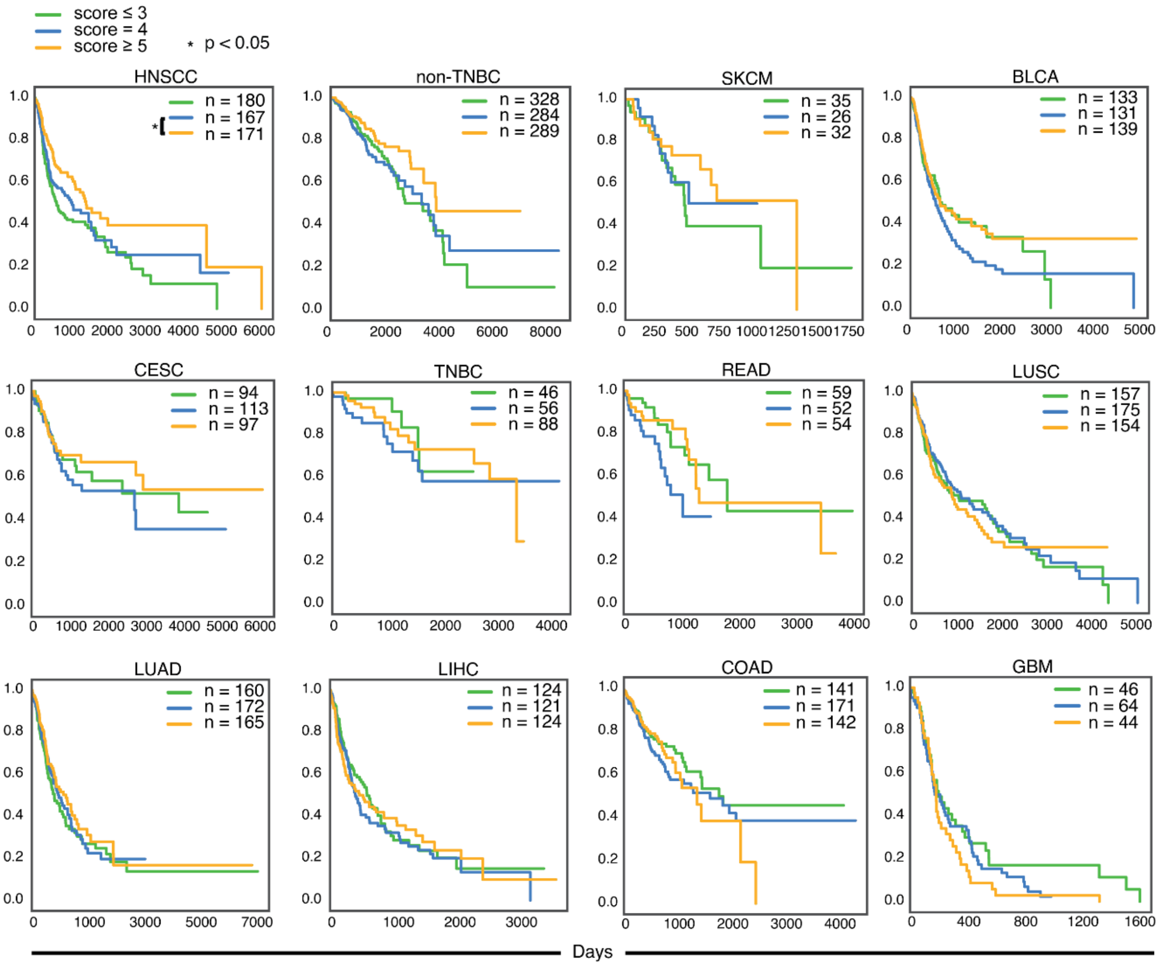

Fig. S1. PFS analysis stratified by the cancer immune score in 11 tumor types. PFS analysis in HNSCC, non-TNBC, SKCM, BLCA, CESC, TNBC, READ, LUSC, LUAD, LIHC, COAD, GBM. BRCA is split into TNBC and non-TNBC. Statistical significance is tested between score ≥5 vs. score = 4, and score = 4 vs. score ≤3.

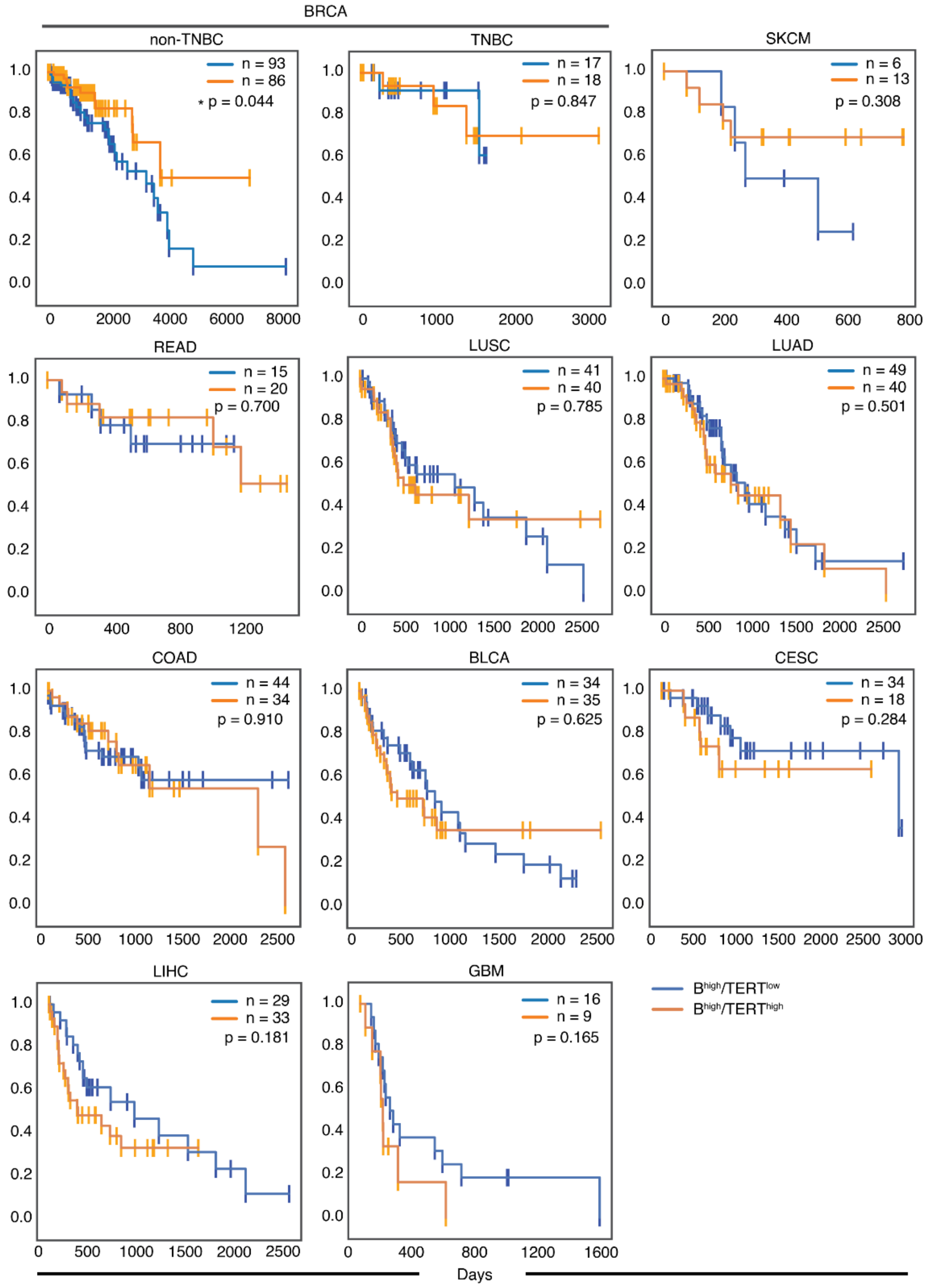

Fig. S2. PFS analysis between B^{high}/TERT^{high} vs B^{high}/TERT^{low} in 10 solid cancer types (excluding HNSCC).

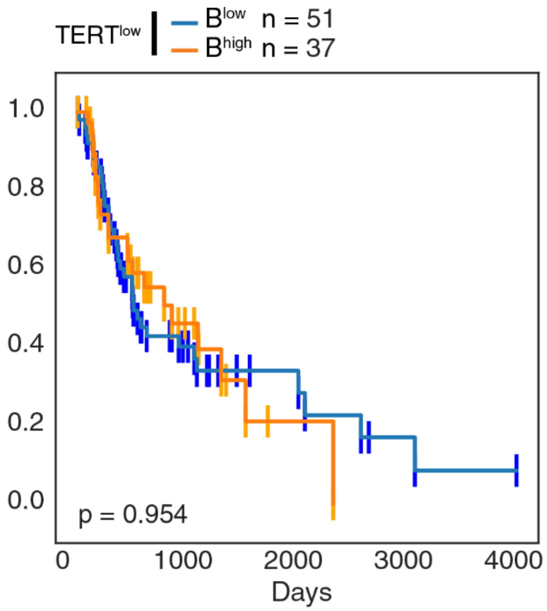

Fig. S3. Progression Free Survival (PFS) analysis of B^{high}/$TERT^{low}$ vs. B^{low}/$TERT^{low}$ in HNSCC.

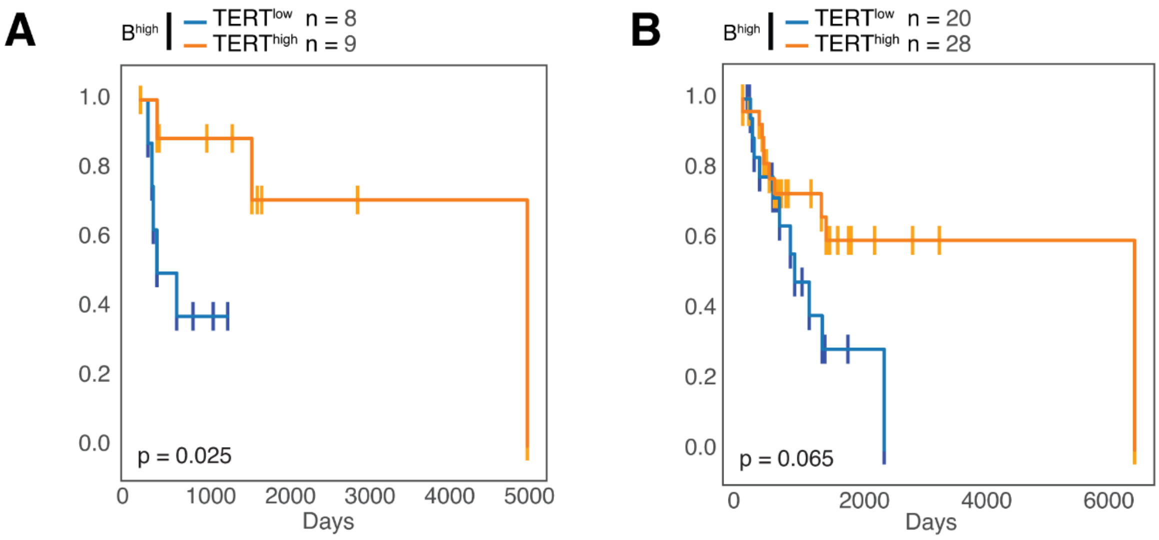

Fig. S4. Progression Free Survival (PFS) analysis of B^{high}/$TERT^{low}$ vs. B^{high}/$TERT^{high}$ for HNSCC tumors, grouped by tumor stages. The group of Stage I-II is shown in (A), and the group of Stage III-IV in (B).

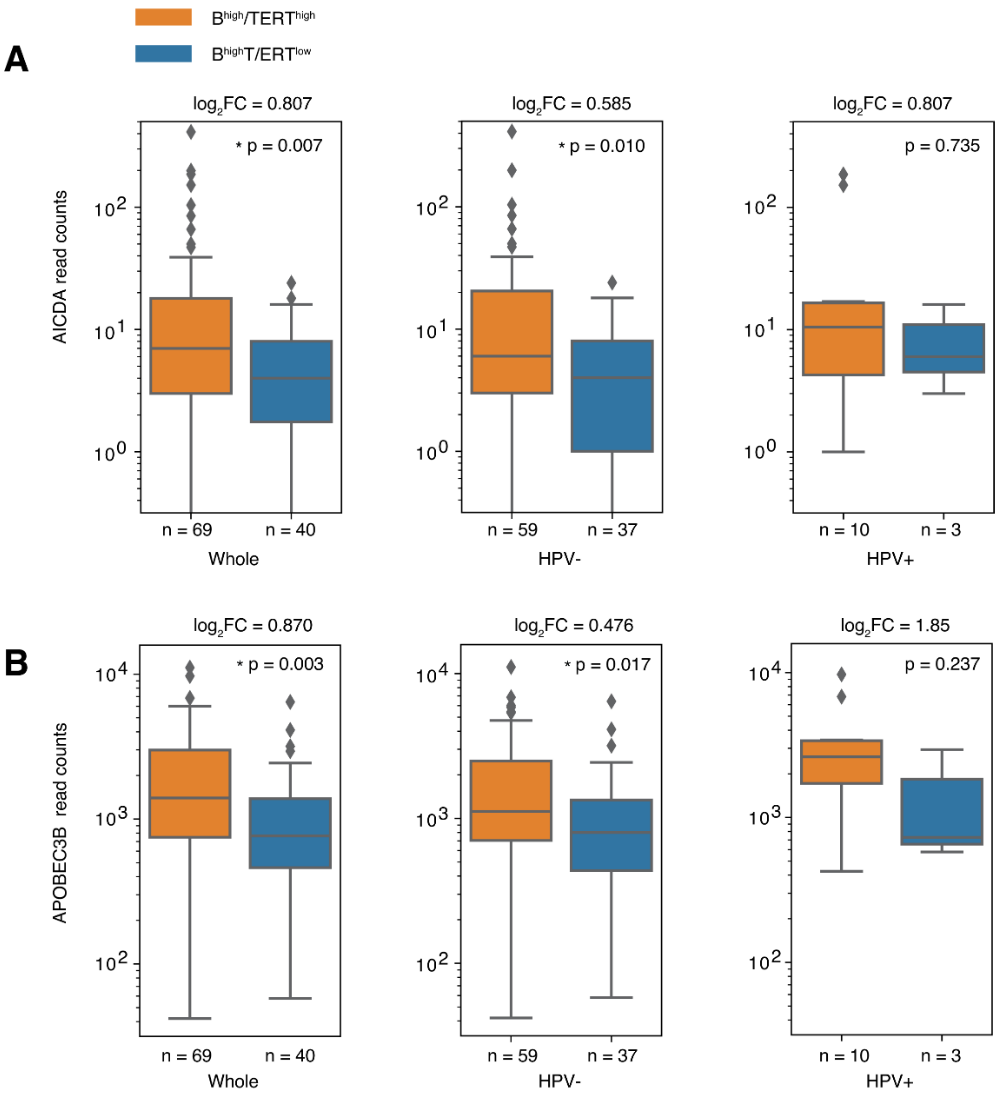

Fig. S5. Expression of AICDA and APOBEC3B in $B^{high}/TERT^{high}$ vs $B^{high}/TERT^{low}$ HNSCC tumors. **(A)** AICDA read count comparison between $B^{high}/TERT^{high}$ vs $B^{high}/TERT^{low}$ in (left) all HNSCC patients, (middle) HPV- HNSCC patients and (right) HPV+ HNSCC patients. Statistical significance is calculated using the Wilcoxon rank-sum test. **(B)** APOBEC3B read count comparison between $B^{high}/TERT^{high}$ vs $B^{high}/TERT^{low}$ in (left) all HNSCC patients, (middle) HPV- HNSCC patients and (right) HPV+ HNSCC patients. Statistical significance is calculated using the Wilcoxon rank-sum test.

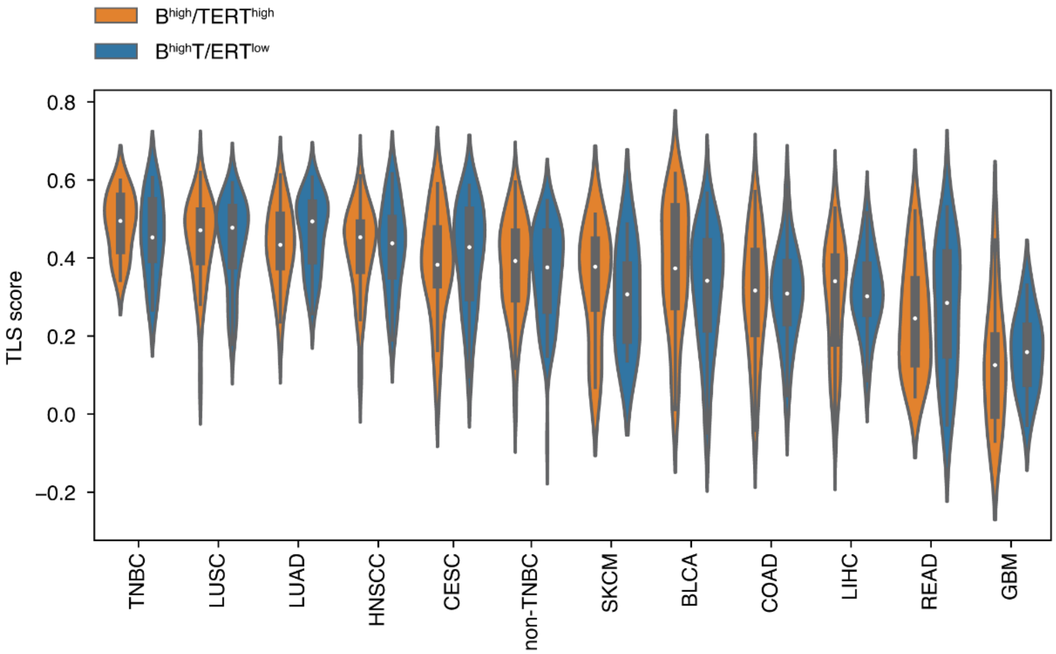

Fig. S6. Comparison of a 12 cytokine-based TLS score across 11 solid cancer types in $B^{high}/TERT^{high}$ vs $B^{high}/TERT^{low}$ groups. TLS score across 11 cancer types comparing $B^{high}/TERT^{high}$ and $B^{high}/TERT^{low}$ ranked by median expression. No differences were statistically significant according to the Wilcoxon rank-sum test.

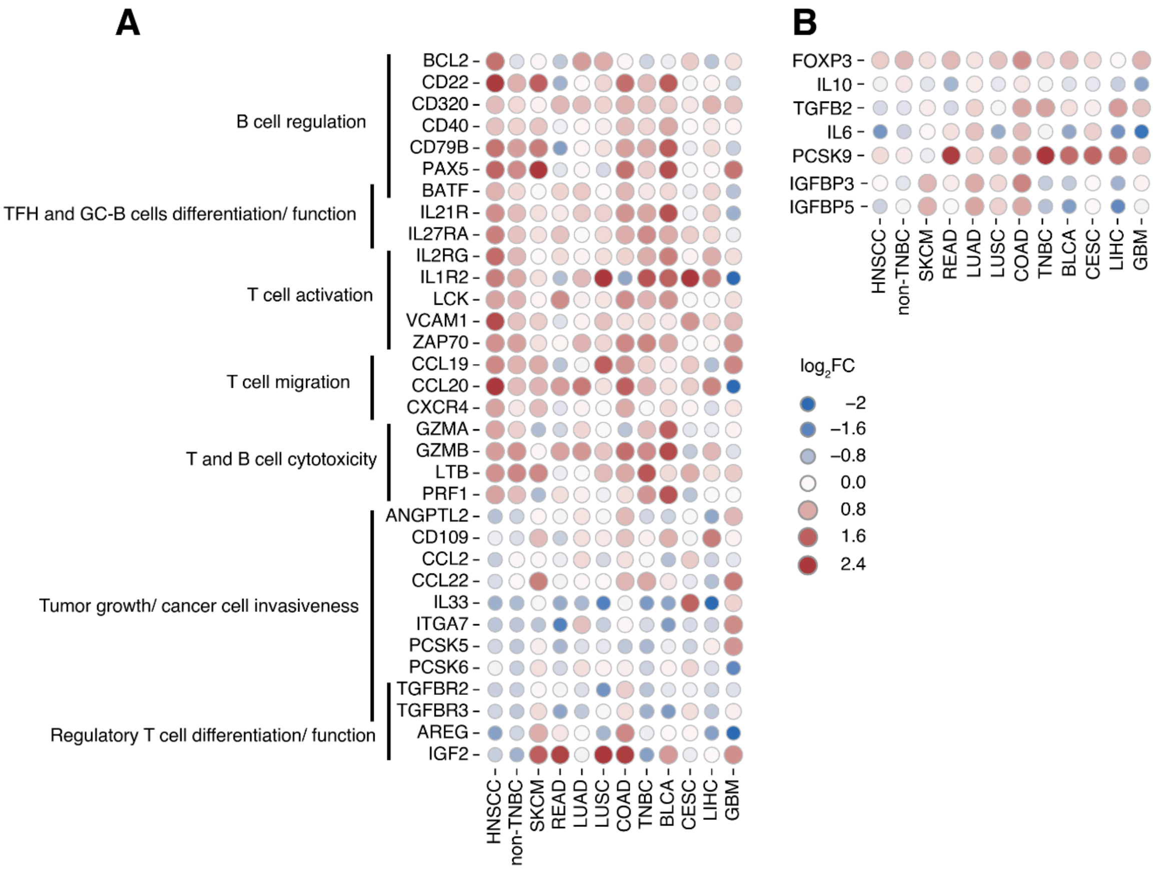

Fig. S7. Differential expression of genes related to several immunological functions in B^{high}/TERT^{high} vs B^{high}/TERT^{low} tumors from 11 solid cancer types. **(A)** Heatmap showing genes found differentially up-regulated and down-regulated genes in HNSCC and grouped by immune pathways across 11 cancer types. Colors and circle size are used to indicate log2FC of the differentially expressed genes between B^{high}/TERT^{high} vs B^{high}/TERT^{low} tumors. **(B)** Similar heatmap for other genes commonly associated with immune suppression and cancer cell invasiveness.

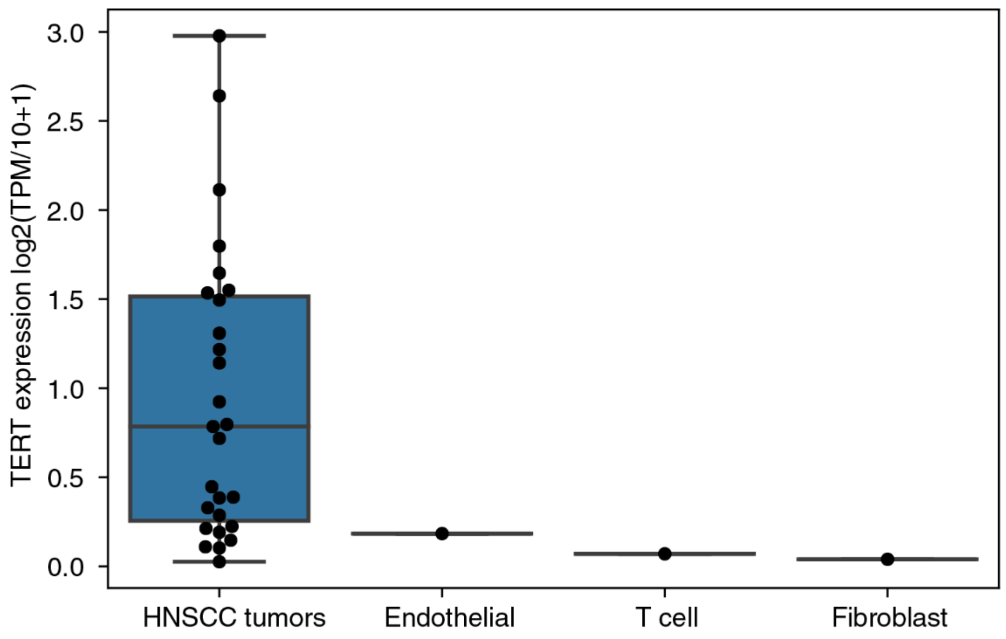

Fig. S8. Boxplot summarization of the distribution of TERT expression from a single-cell RNAseq study of HNSCC tumors. TERT expression levels are shown for the cell types and cells that are greater than zero TERT expression, including tumor cells (n=27) infiltrated endothelial cells (n = 1), T cells (n = 1), and Fibroblasts (n = 1).

Table S1. Cox proportional hazard model coefficient for B cells in TERT^{low} and TERT^{high}

A.

	Coeff.	Coeff. lower 95%	Coeff. upper 95%	z	p
**Age**	-0.00	-0.03	0.02	-0.16	0.88
**Sex**	0.09	-0.53	0.71	0.29	0.77
**B cell**	0.08	-0.29	0.46	0.44	0.66
**logTMB**	0.06	-0.26	0.39	0.38	0.70
**Stage**	0.21	-0.07	0.50	1.48	0.14
**HPV+**	-0.15	-1.07	0.77	-0.32	0.75

B.

	Coeff.	Coeff. lower 95%	Coeff. upper 95%	z	p
**Age**	0.00	-0.03	0.02	-0.20	0.84
**Sex**	0.05	-0.55	0.65	0.16	0.87
**B cell**	0.08	-0.31	0.47	0.40	0.69
**NeoAg**	0.01	-0.04	0.03	-0.43	0.67
**stage**	0.19	-0.09	0.48	1.34	0.18
**HPV+**	0.18	-1.10	0.74	-0.39	0.70

C.

	Coeff.	Coeff. lower 95%	Coeff. upper 95%	z	p
**Age**	0.02	-0.01	0.04	1.13	0.26
**Sex**	-0.20	-0.86	0.45	-0.61	0.54
**B cell**	-0.40	-0.77	-0.03	-2.11	0.04
**logTMB**	-0.08	-0.54	0.39	-0.32	0.75
**Stage**	0.28	-0.06	0.61	1.63	0.10
**HPV+**	-0.24	-1.15	0.67	-0.52	0.60

D.

	Coeff.	Coeff. lower 95%	Coeff. upper 95%	z	p
**Age**	0.02	-0.01	0.05	1.26	0.21
**Sex**	-0.20	-0.86	0.46	-0.58	0.56
**B cell**	-0.44	-0.81	-0.06	-2.27	0.02
**NeoAg**	-0.05	-0.12	0.03	-1.27	0.20
**Stage**	0.29	-0.05	0.63	1.70	0.09
**HPV+**	-0.29	-1.19	0.62	-0.62	0.53

A. Cox proportional hazard model for B cells, including age, sex, tumor mutational burden (TMB), HPV positivity, and tumor stages as covariates, limiting our analysis to the $TERT^{low}$ population in HNSCC (n = 150).

B. Cox proportional hazard model for B cells, including age, sex, neoantigen level, HPV positivity, and tumor stages as covariates, limiting our analysis to the $TERT^{low}$ population in HNSCC (n = 146).

C. Cox proportional hazard model for mutational burden (TMB), B cells, including age, sex, tumor, HPV positivity, and tumor stages as covariates, limiting our analysis to the $TERT^{high}$ population in HNSCC (n = 150).

D. Cox proportional hazard model for neoantigen level including B cells, age, sex, HPV positivity, and tumor stages as covariates, limiting our analysis to the $TERT^{high}$ population in HNSCC (n = 146).

Table S2. Cox proportional hazard model coefficient for conserved antigens

	Coeff.	Coeff. lower 95%	Coeff. upper 95%	z	p
**CTAG1B**	-1.02	-3.08	1.03	-0.98	0.33
**MUC1**	0.00	0.00	0.01	1.28	0.20
**MAGEA4**	0.00	-0.01	0.00	-1.06	0.29
**MAGEA3**	-0.01	-0.02	0.00	-2.00	0.05
**CEACAM5**	0.00	0.00	0.00	-0.92	0.36

The Table summarizes coefficients of conserved antigens from Cox proportional hazard models fitted for each conserved antigen independently, including age, sex, HPV positivity, and tumor stage as covariates, and limiting our analysis to the TERT^{low} population (n = 150) in HNSCC.

Table S3. Gene signatures for the detection of tertiary lymphoid structures in human cancers

	Signature	Gene names	Reference
**TLS-1**	TFH cell signature	CXCR5, ICOS, CD40LG, PDCD1, CXCL13	*(40)*
**TLS-2**	CIBERSORT TFH gene signature (top 20 genes)	TRAC, CD3D, TRBC1, CD2, LTB, LCK, CXCL13, ITK, LAT, ICOS, PASK, ZAP70, FAIM3, IL2RB, CD69, TRIB2, FOSB, CD27, PTPRCAP, CD247, SIK1	*(39)*
**TLS-3**	12-Chemokine signature	CCL2, CCL3, CCL4, CCL5, CCL8, CCL18, CCL19, CCL21, CXCL9, CXCL10, CXCL11, CXCL13	(13)
**TLS-4**	TFH cell signature	CXCL13, CD200, FBLN7, ICOS, SGPP2, SH2D1A, TIGIT, PDCD1	(13)
**TLS-5**	TH1 cell and B cell signature	CD4, CCR5, CXCR3, CSF2, IGSF6, IL2RA, CD38, CD40, CD5, MS4A1	(13)

Table S4. Expression of TERT in log_2 TPM for 10 immune cell types

**Immune cell types**	**log_2TPM**
B cells	0.0
Plasma cells	0.694
T cells CD8	0.144
T cells CD4	1.539
NK cells	0.0
Monocytes	0.0
Dendritic cells	0.0
Mast cells	0.015
Eosinophils	0.0
Neutrophils	0.0

The expression of TERT in log_2 TPM for 10 immune cell types was calculated using CIBERSORTx applied to TCGA HNSCC data.
